# A Cardiologia Intervencionista Brasileira e as Oclusões Coronárias Crônicas: Onde Estamos?

**DOI:** 10.36660/abc.20230172

**Published:** 2023-04-18

**Authors:** José Mariani

**Affiliations:** 1 Hospital Israelita Albert Einstein São Paulo SP Brasil Hospital Israelita Albert Einstein, São Paulo, SP – Brasil; 2 Santa Casa de Misericórdia de São Paulo São Paulo SP Brasil Santa Casa de Misericórdia de São Paulo, São Paulo, SP – Brasil; 3 Faculdade de Ciências Médicas Santa Casa de São Paulo São Paulo SP Brasil Faculdade de Ciências Médicas da Santa Casa de São Paulo, São Paulo, SP – Brasil

**Keywords:** Oclusão Coronária/mortalidade, Oclusão Coronária/terapia, Stents Farmacológicos, Ultrasonografia, Intervencional/métodos, Angiografia Coronária/métodos, Diagnóstico por Imagem/métodos

A doença aterosclerótica coronária tem várias facetas de apresentação clínica, sendo a oclusão coronária crônica (OCC), aquela que produz obstrução total da luz do vaso conhecida ou presumida com três meses ou mais. Está presente em 16-18% dos pacientes que possuem doença coronária significativa.^1^

O tratamento percutâneo deste tipo de lesão é quase tão antigo quanto a primeira angioplastia coronária realizada por Andreas Gruntzig em 1979. Foi realizada pela primeira vez por Martin Kaltenbach em Frankfurt no final da década de 70 e por Geoffrey Hartzler em Kansas City nas décadas seguintes.^2 , 3^ Desde então, a crescente e espetacular incorporação de tecnologia à medicina, especialmente na Cardiologia Intervencionista, atrelada ao conhecimento e expertise de novas técnicas dedicadas às intervenções coronárias complexas, onde estão incluídas as OCC, permitiram grande, seguro e efetivo avanço na abordagem destas lesões.

Existem registros, documentos, artigos e consensos internacionais dedicados a este cenário.^4 - 6^ Conhecer os dados nacionais se faz de extrema importância, pois nos posiciona perante a comunidade internacional. Também possibilita o desenvolvimento de medidas assertivas e efetivas e programas dedicados à educação médica continuada e o desenvolvimento de políticas tanto na esfera pública quanto na saúde suplementar para este tipo de procedimento, o qual requer não só conhecimento técnico, mas também dispositivos dedicados de alto custo, nem sempre disponíveis.

No artigo aqui publicado por Botelho da Silva, et al.^7^ “Panorama das intervenções coronarianas percutâneas em oclusões totais crônicas em centros participantes do LATAM CTO REGISTRY no Brasil”, temos a grata felicidade de observar que nosso país está muito bem posicionado e representado frente às mais importantes instituições do mundo que se dedicam ao tratamento destas obstruções, com similares taxas de sucesso técnico (84% dos procedimentos), eventos adversos (2,3%) e de mortalidade (0,75%). Este primeiro relato da prática médica acerca da recanalização das OCC em 26 centros no Brasil mostra também um fator primordial para nossa prática clínica diária, que é a excepcional aderência às indicações clínicas para este tipo de procedimento: cerca de 95% dos pacientes apresentavam ou angina limitante ou isquemia moderada/severa documentada.

Cabe aqui registrar, não só para este registro bem como para todos os outros dedicados à esta estratégia, a baixa utilização de imagem intravascular, principalmente do ultrassom intracoronário, utilizado em 10% dos casos desta série, provavelmente por problemas de reembolso tanto na esfera pública quanto privada. Estudos^8^ têm demonstrado uma redução significativa de eventos combinados (morte e infarto) e da revascularização do vaso alvo quando esta ferramenta é utilizada com o objetivo de otimizar os resultados da intervenção e também para auxiliar na identificação e localização da capa proximal quando esta é ambígua, bem como nas técnicas de dissecção-reentrada, aumentando as taxas de sucesso deste procedimento.

A Cardiologia Intervencionista Brasileira continua sendo equiparada aos maiores e melhores centros mundiais, a despeito de toda e qualquer limitação que porventura possa existir em nosso complexo sistema de reembolso, quer seja ele público ou privado.

Boa leitura a todos.
