# Supplementary material for: A Cardiologia Intervencionista Brasileira e as Oclusões Coronárias Crônicas: Onde Estamos?
Source: Arq Bras Cardiol. 2023 Apr 18;120(4):e20230172. [Article in Portuguese] doi: 10.36660/abc.20230172 (PMC10263424; doi:10.36660/abc.20230172)
Supplement: Translation

# Translation: Brazilian Interventional Cardiology and Chronic Coronary Occlusions: Where Are We?

**Authors:** José Mariani

**Affiliations:** 1 Brasil Hospital Israelita Albert Einstein, São Paulo, SP – Brasil; 2 Brasil Santa Casa de Misericórdia de São Paulo, São Paulo, SP – Brasil; 3 Brasil Faculdade de Ciências Médicas da Santa Casa de São Paulo, São Paulo, SP – Brasil

**Keywords:** Coronary Occlusion/mortality, Coronary Occlusion/therapy, Drug-Eluting Stents, Ultrasonography,Interventional/methods, Coronary Angiography/methods, Diagnostic Imaging/methods

The coronary atherosclerotic disease has several facets of clinical presentation; chronic coronary occlusion (CCO) is one that produces total obstruction of the lumen of the vessel, known or presumed for three months or more. It is present in 16-18% of patients with significant coronary disease.^1^

Percutaneous treatment of this type of lesion is almost as old as the first coronary angioplasty performed by Andreas Gruntzig in 1979. Martin Kaltenbach first performed it in Frankfurt in the late 1970s and by Geoffrey Hartzler in Kansas City in the following decades.^2 , 3^Since then, the growing and spectacular incorporation of technology into medicine, especially in Interventional Cardiology, associated with the knowledge and expertise of new techniques dedicated to complex coronary interventions, which include CCO, have allowed great, safe, and effective advances in the approach to these lesions.

Records, documents, articles, and international consensuses are dedicated to this scenario.^4 - 6^Knowing the national data is extremely important, as it positions us before the international community. It also enables the development of assertive and effective measures and programs dedicated to continuing medical education and the development of policies both in the public sphere and in supplementary health for this type of procedure, which requires not only technical knowledge but also dedicated high-cost devices, not always available.

In the article published here by Botelho da Silva AC et al.^7^“Overview of percutaneous coronary interventions in chronic total occlusions in centers participating in the LATAM CTO REGISTRY in Brazil”, we have grateful happiness to observe that our country is very well positioned and represented in the face of the most important institutions in the world that are dedicated to the treatment of these obstructions, with similar technical success rates (84% of procedures), adverse events (2.3%) and mortality (0.75%). This first report from medical practice about the recanalization of CCO in 26 centers in Brazil also shows a key factor for our daily clinical practice, which is the exceptional adherence to the clinical indications for this type of procedure: about 95% of the patients had either angina or limiting or documented moderate/severe ischemia.

It is worth noting here, not only for this registry as well as for all others dedicated to this strategy, the low use of intravascular imaging, mainly intravascular ultrasound, used in 10% of the cases in this series, probably due to reimbursement problems both in the public sphere how private. Studies^8^have shown a significant reduction in combined events (death and infarction) and target vessel revascularization when this tool is used to optimize intervention results and also to help identify and locate the proximal layer when it is ambiguous, as well as in dissection-reentry techniques, increasing the success rates of this procedure.

Brazilian Interventional Cardiology continues to be equated with the largest and best centers in the world, despite any and all limitations that may exist in our complex reimbursement system, whether public or private.

Happy reading to everyone.
